# Sleep disorders in adults treated at Health and Community Action Center No. 27, Buenos Aires, Argentina

**DOI:** 10.1192/j.eurpsy.2026.12086

**Published:** 2026-07-31

**Authors:** C. B. Wydler, E. Sarcona, C. Fraga, A. azcarate

**Affiliations:** 1 Psychiatry; 2 Medical doctor, Health and Community Action Center No. 27, Pirovano Hospital; 3 Medicine, University of Buenos Aires, Buenos Aires, Argentina

## Abstract

**Introduction:**

Poor sleep quality is a common problem in the population and can negatively affect both physical and mental health.

**Objectives:**

To evaluate the Pittsburgh Sleep Quality Index (PSQI) in a population aged 18 years or older attending Health and Community Action Center No. 27 in the City of Buenos Aires, Argentina.

**Methods:**

This was an observational, analytical, prospective, cross-sectional study. The Pittsburgh Sleep Quality Questionnaire was administered to 208 patients in a targeted manner to assess the quantity and quality of their sleep.

**Results:**

The 208 patients interviewed had a mean age of 48.3 ± 14.6 years, and 160 (76.9%) were women. They slept an average of 6.3 ± 1.5 hours per night, and the results for sleep quality were: latency > 15 minutes 81.3% (169p), daytime dysfunction 66.3% (138p), duration < 7 hours 58.7% (122p), efficiency < 85% 57.2% (119p), use of hypnotics 27.4% (57p), reported poor or very poor sleep quality 27% (77p), and sleep disturbances 15.9% (33p). The PSQI > 5, reflecting poor sleep quality, was found in 65.4% (136p).

**Conclusions:**

Given the high prevalence of poor sleep quality in the population treated, it would be important to routinely assess patients’ sleep quality in consultations and offer treatment or inform them. Although cognitive behavioral therapy for insomnia (CBT) is considered the first line of treatment for patients with chronic insomnia, psychotropic medications are frequently prescribed in psychiatry as the first line of treatment for patients with this problem. In cases where non-pharmacological measures are implemented, they generally only include sleep-related dietary measures in combination with hypnotics or other medications. However, other CBT tools are not usually provided.

**Disclosure of Interest:**

None Declared

